# Relationship between HER2 expression and efficacy with first-line trastuzumab emtansine compared with trastuzumab plus docetaxel in TDM4450g: a randomized phase II study of patients with previously untreated HER2-positive metastatic breast cancer

**DOI:** 10.1186/bcr3661

**Published:** 2014-05-23

**Authors:** Edith A Perez, Sara A Hurvitz, Lukas C Amler, Kirsten E Mundt, Vivian Ng, Ellie Guardino, Luca Gianni

**Affiliations:** 1 Mayo Clinic, 4500 San Pablo Road, Jacksonville, FL 32224, USA; 2 UCLA Jonsson Comprehensive Cancer Center and Translational Research in Oncology-US, Los Angeles, CA, USA; 3 Genentech, Inc, South San Francisco, CA, USA; 4 Department of Medical Oncology, San Raffaele Hospital, Milan, Italy

## Abstract

**Introduction:**

The purpose of this study was to retrospectively explore the relationship between human epidermal growth factor receptor 2 (HER2) messenger RNA (mRNA) expression and efficacy in patients receiving trastuzumab plus docetaxel (HT) or trastuzumab emtansine (T-DM1).

**Methods:**

Patients with HER2-positive, locally advanced or metastatic breast cancer (MBC) were randomly assigned to HT (*n* = 70) or T-DM1 (*n* = 67). HER2 status was assessed locally using immunohistochemistry or fluorescence in situ hybridization and confirmed retrospectively by central testing. HER2 mRNA expression was assessed using quantitative reverse transcriptase polymerase chain reaction.

**Results:**

HER2 mRNA levels were obtained for 116/137 patients (HT = 61; T-DM1 = 55). Median pretreatment HER2 mRNA was 8.9. The risk of disease progression in the overall population was lower with T-DM1 than with HT (hazard ratio (HR) = 0.59; 95% confidence interval (CI) 0.36 to 0.97). This effect was more pronounced in patients with HER2 mRNA ≥ median (HR = 0.39; 95% CI 0.18 to 0.85) versus < median (HR = 0.85; 95% CI 0.44 to 1.67). In the T-DM1 arm, median progression-free survival (PFS) was not reached in patients with HER2 mRNA ≥ median and was 10.6 months in patients with HER2 mRNA < median. In the HT arm, PFS was 8.8 versus 9.8 months in patients with HER2 mRNA ≥ median versus < median, respectively. The effect of HER2 mRNA expression on objective response rates was less pronounced.

**Conclusions:**

This exploratory analysis suggests that while overall, patients with HER2-positive MBC show improved PFS with T-DM1 relative to HT, the effect is enhanced in patients with tumor HER2 mRNA ≥ median.

**Trial registration:**

ClinicalTrials.gov NCT00679341

## Introduction

Adding human epidermal growth factor receptor 2 (HER2)-targeted agents such as trastuzumab, trastuzumab and pertuzumab, or lapatinib to chemotherapy can improve clinical outcomes for patients with HER2-positive metastatic breast cancer (MBC) [1-5]. Despite receiving treatment with these agents, however, patients will eventually develop disease progression, and novel HER2-directed therapies are still needed.

Trastuzumab emtansine (T-DM1), which was recently approved as a single agent for the treatment of HER2-positive MBC, is an antibody-drug conjugate comprising trastuzumab stably linked to DM1, which is a potent cytotoxic agent derived from the antimicrotubule agent maytansine [6]. The trastuzumab component of T-DM1 targets the agent to HER2-expressing tumor cells, within which it is internalized and lysine-N^ϵ^-4 -MCC-DM1 is released intracellularly, resulting in microtubule instability and apoptosis [6,7]. Like trastuzumab, T-DM1 also induces antibody-dependent cellular cytotoxicity, inhibits HER2 pathway proliferative signaling, and inhibits HER2 shedding [8]. In the pivotal phase III EMILIA trial (NCT00829166), patients with pretreated (trastuzumab and a taxane), HER2-positive locally advanced or MBC were randomly assigned to T-DM1 or capecitabine plus lapatinib [9]. Patients who received T-DM1 had significant improvements in both progression-free survival (PFS) and overall survival (OS) compared with those in the comparator arm [9]. Significant improvements in PFS were also reported in the randomized phase II TDM4450g (BO21976; NCT00679341) study, in which patients with previously untreated MBC received either trastuzumab plus docetaxel (HT) or T-DM1 [10].

Results from exploratory analyses of single-arm, phase II studies of T-DM1 in patients with MBC (TDM4258g and TDM4374g) suggest that greater expression of HER2 messenger RNA (mRNA), which was assessed using quantitative reverse transcriptase polymerase chain reaction (qRT-PCR), may correlate with greater objective response rates (ORRs) and longer PFS [11,12]. Here we report the results from an exploratory analysis of data from the TDM4450g (BO21976) study, to examine whether HER2 expression (assessed by qRT-PCR) correlates with clinical benefit reported in patients receiving treatment with T-DM1 in this study.

## Materials and methods

### Patients

Eligible patients were ≥18 years of age with unresectable, HER2-positive, locally advanced MBC and/or MBC that had not been treated with prior chemotherapy (prior hormonal therapy was allowed). Patients had measurable disease, as assessed by the response evaluation criteria in solid tumors [13], an Eastern Cooperative Oncology Group (ECOG) performance status (PS) of 0 or 1, and adequate organ function. Detailed information on the methodology employed in this study has been published elsewhere [10].

### Study design

In this phase II, multicenter, open-label study, patients were randomly assigned (1:1 ratio) to T-DM1 (3.6 mg/kg intravenous (IV) every 3 weeks), or trastuzumab (8 mg/kg IV on cycle 1, day 1, followed by 6 mg/kg IV every 3 weeks) plus docetaxel (75 mg/m^2^ or 100 mg/m^2^ IV). Treatments were given until disease progression, unacceptable toxicity, or study closure. The primary efficacy end point was investigator-assessed PFS. A number of exploratory end points were also investigated, including the relationship between the levels of HER2 mRNA expression and efficacy outcomes.

All participating study sites had institutional review board (IRB) approval of the protocol (see list of IRBs in Additional file 1). The study was conducted in accordance with the Declaration of Helsinki, the US Food and Drug Administration Good Clinical Practices, and local laws. All patients provided written informed consent.

### HER2 quantification

Tumor HER2 status was established by local laboratory testing, using either immunohistochemistry (IHC; positive result: 3+ staining intensity in >10% of cells) or fluorescence in situ hybridization (FISH; positive result: HER2/chromosome 17 centromere ratio ≥2.0). Archival tumor tissue (paraffin block and/or slides) was also sent for central confirmation of HER2 status using FISH and IHC at Targos Molecular Pathology GmbH (Kassel, Germany), but central HER2 testing was not required for initiation of treatment. HER2 protein expression was assessed using the HercepTest™ kit (Dako North America, Inc., Carpinteria, CA, USA). *HER2* gene amplification was assessed using the standard PathVysion HER2 FISH kit (Abbott Molecular, Abbott Park, IL, USA). Both methods were carried out in accordance with the manufacturers’ instructions and with the instructions in the trastuzumab prescribing information [14].

Samples that were sent for central HER2 testing were also used for analysis of HER2 mRNA expression by qRT-PCR at the same central laboratory. To allow for analysis of mRNA as a continuous variable, mRNA expression was measured in samples from all randomly assigned patients regardless of the HER2 status established by central review. RNA was extracted and analyzed using the LightCycler® (Roche Applied Sciences, Mannheim, Germany) in accordance with the manufacturer’s instructions. The HER2 mRNA values obtained were relative to the housekeeping gene *Glucose-6-phosphate dehydrogenase*.

### Assessments

Methods used for tumor assessments and clinical outcomes have been published previously [10].

### Statistical analysis

This study had a hypothesis-generating statistical design. The study sponsor collected and analyzed the data, authors were involved in the study design, and all authors had access to the primary data. Demographic variables and baseline characteristics were summarized by treatment arm and by treatment arm and HER2 mRNA expression. Pretreatment HER2 mRNA values were summarized by treatment arm. The hazard ratio (HR) of PFS comparing T-DM1 with HT and its 95% CI was estimated from a Cox proportional hazards model by HER2 mRNA subgroups. Kaplan-Meier estimates of PFS and median PFS were presented by HER2 mRNA subgroups. An estimate of the ORR was calculated for each treatment arm by HER2 mRNA subgroups.

Multivariate Cox regression analysis was performed to estimate treatment effect adjusting for multiple prognostic baseline covariates. The variables tested in the model included age, race, world region, ECOG PS, progesterone receptor (PR) and estrogen receptor (ER) status, central HER2 status, number of disease sites, disease measurability, disease-free interval, disease stage at initial diagnosis, menopausal status, prior anthracycline, prior taxane, prior trastuzumab, prior taxane and trastuzumab, prior taxane or trastuzumab, lung or liver involvement, prior hormonal therapy, prior radiotherapy, and HER2 mRNA (below the median versus equal to or higher than the median for the overall population). A stepwise procedure was used to determine the final model.

## Results

The database lock for the primary efficacy analysis took place on 15 November 2010 after 75 investigator-assessed PFS events had taken place in the two treatment arms combined, as pre-specified in the statistical analysis plan of the study.

### Patient characteristics

In total, 137 patients were randomly assigned to treatment with either HT (n = 70) or T-DM1 (n = 67) (see Additional file 2 for CONSORT diagram). Baseline characteristics were similar between the treatment arms (Table 1), with the exception that more patients in the HT arm were first diagnosed at an earlier disease stage (stage I to III at diagnosis was 68.1% with HT versus 58.2% with T-DM1). Consistent with this, more patients in the HT arm had received prior (neo) adjuvant therapy with trastuzumab (27.1% versus 17.9%) or a taxane (40.0% versus 32.8%).

**Table 1:** Selected patient demographic and baseline characteristics by treatment arm

**Characteristic**	**HT (n = 70)**	**T-DM1 (n = 67)**
**Median age, years (range)**	52.0 (33 to 75)	55.0 (27 to 82)
**World region, %**		
North America	28.6	31.3
Central and South America	28.6	23.9
Europe	42.9	44.8
**Race, %**		
White	82.9	77.6
American Indian or Alaskan native	10.0	7.5
Black	4.3	4.5
Other or not available	2.9	10.4
**ECOG PS, %**		
0	63.8^a^	65.7
1	36.2^a^	34.3
**HER2 status by central laboratory, %**^**b**^		
HER2-positive	85.9	85.7
Normal	14.1	14.3
**ER/PR status, %**		
ER-positive and/or PR-positive	54.3	49.3
ER-negative and PR-negative	41.4	47.8
ER and PR unknown	4.3	3.0
**Stage at initial diagnosis, %**		
Stage I to III	68.1^a^	58.2
Stage IV	29.0^a^	34.3
Unknown	2.9^a^	7.5
**Number of distinct sites of involvement**		
1 to 2	49.3^a^	35.8
>2	50.7^a^	64.2
**Lung or liver involvement. %**		
Yes	67.1	71.6
No	31.4	26.9
Unknown	1.4	1.5
**Disease-free interval, %**		
≤24 months	64.3	59.7
>24 months	35.7	40.3
**Prior treatment. %**		
Trastuzumab	27.1	17.9
Taxane	40.0	32.8
Anthracycline	48.6	44.8
**Total number of prior chemotherapy agents, median (range)**	3 (1 to 4)	3 (1 to 6)

Reproduced with permission from Hurvitz *et al*. [10]. ^a^Data were available for 69 patients in the HT arm. ^b^Central testing for HER2 status was performed for 64 patients in the HT arm and 63 patients in the T-DM1 arm. ECOG PS, Eastern Cooperative Oncology Group performance status; ER, estrogen receptor; HER2, human epidermal growth factor receptor 2; HT, trastuzumab plus docetaxel; PR, progesterone receptor; T-DM1, trastuzumab emtansine.

### Treatment

Details of treatment duration and discontinuation have been published previously [10].

### HER2 quantification

Of the 137 randomized patients, 127 (63 in the T-DM1 arm; 64 in the HT arm) had tumor samples analyzed centrally for confirmatory HER2 testing. Testing was performed on the primary tumor in 89 patients, on a metastatic lesion in 12 patients, and the status of the tumor (that is, primary or metastatic) was not reported or was unknown in 36 patients. At the time of this analysis 85.7% of patients in the T-DM1 arm and 85.9% of patients in the HT arm had confirmed centrally tested HER2-positive primary tumors. Valid results for the assessment of pretreatment HER2 mRNA were obtained for 116 patients, with 55 in the T-DM1 arm and 61 in the HT arm. Results for six samples were below the limit of quantification, and there was not enough tissue available to run the analyses for five patients. The median (range) pretreatment levels of HER2 mRNA in the overall patient population were 8.9 (0.4 to 105.0; units used were the concentration ratio). Median (range) pretreatment levels of HER2 mRNA were 10.3 (0.4 to 103.0) in the T-DM1 arm and 8.7 (0.5 to 105.0) in the HT arm.

Baseline patient and tumor characteristics were generally similar between patients with HER2 mRNA expression equal to or greater than the median (that is, the median of the overall study population) and below the median (that is, the median of the overall study population), regardless of the treatment arm (Table 2) with some exceptions. A greater proportion of patients in the subgroup with HER2 mRNA expression equal to or greater than the median had centrally confirmed HER2 status (100% versus 72.4%). In addition, a greater proportion of patients with median tumor expression of HER2 mRNA equal to or greater than the median versus below the median were from outside of the US (74.1% versus 63.8%), had ER-negative and/or PR-negative tumors (50.0% versus 34.5%), and equal to or fewer than two sites of disease involvement (47.4% versus 37.9%).

**Table 2:** Selected patient demographic and baseline characteristics by HER2 mRNA expression and treatment arm

**Characteristic**	**HER2 mRNA < median (n = 58)**			**HER2 mRNA ≥ median (n = 58)**		
	**HT**	**T-DM1**	**Total**	**HT**	**T-DM1**	**Total**
	**(n = 32)**	**(n = 26)**	**(n = 58)**	**(n = 29)**	**(n = 29)**	**(n = 58)**
**Median age, years (range)**	52.5	54.5	53.5	51.0	54.0	53.0
	(36 to 72)	(32 to 74)	32 to 74	(33 to 75)	(27 to 82)	(27 to 82)
**World region, %**						
US	34.4	38.5	36.2	24.1	27.6	25.9
Non-US	65.6	61.5	63.8	75.9	72.4	74.1
**Race, %**^**a**^						
White	81.3	80.8	81.0	86.2	72.4	79.3
American Indian or Alaskan native	9.4	7.7	8.6	6.9	6.9	6.9
Black	6.3	0.0	3.4	3.4	6.9	5.2
Other or not available	3.1	11.5	6.9	3.4	13.8	8.6
**ECOG PS, %**						
0	62.5	80.8	70.7	57.1	69.0	63.2
1	37.5	19.2	29.3	42.9	31.0	36.8
**HER2 status by central laboratory, %**						
HER2-positive	71.9	73.1	72.4	100.0	100.0	100.0
Normal	28.1	26.9	27.6	0.0	0.0	0.0
**ER/PR status, %**^**a**^						
ER-positive and/or PR-positive	65.6	57.7	62.1	48.3	44.8	46.6
ER-negative and PR-negative	28.1	42.3	34.5	48.3	51.7	50.0
ER and PR unknown	6.3	0	3.4	3.4	3.4	3.4
**Number of distinct sites involved**^**a**^						
1 to 2	43.8	30.8	37.9	50.0	44.8	47.4
>2	56.3	69.2	62.1	50.0	55.2	52.6

^a^Percentages may not add to 100% due to rounding. ECOG PS, Eastern Cooperative Oncology Group performance status; ER, estrogen receptor; HER2, human epidermal growth factor receptor 2; HT, trastuzumab plus docetaxel; mRNA, messenger RNA; PR, progesterone receptor; T-DM1, trastuzumab emtansine.

Among patients with HER2 mRNA expression equal to or greater than the median, a greater proportion of patients in the T-DM1 arm relative to the HT arm had ECOG PS of 0 (69.0% versus 57.1%) and more patients in the HT arm were white (86.2% versus 72.4%) (Table 2). Among patients with HER2 mRNA expression below the median, a greater proportion of patients in the T-DM1 arm relative to the HT arm had ECOG PS 0 (80.8% versus 62.5%), ER-negative/PR-negative tumors (42.3% versus 28.1%), and more than two sites of disease involvement (69.2% versus 56.3%).

### Clinical outcomes by HER2 expression

The PFS benefit with T-DM1 in patients with centrally confirmed HER2-positive tumors (median PFS in the T-DM1 arm versus the HT arm 14.2 months versus 9.8 months; HR for PFS 0.57, 95% CI 0.32, 1.04) was similar to that seen in the overall study population (median PFS in the T-DM1 arm versus the HT arm 14.2 months versus 9.2 months; HR for PFS 0.59, 95% CI 0.36, 0.97).

**Figure 1:**
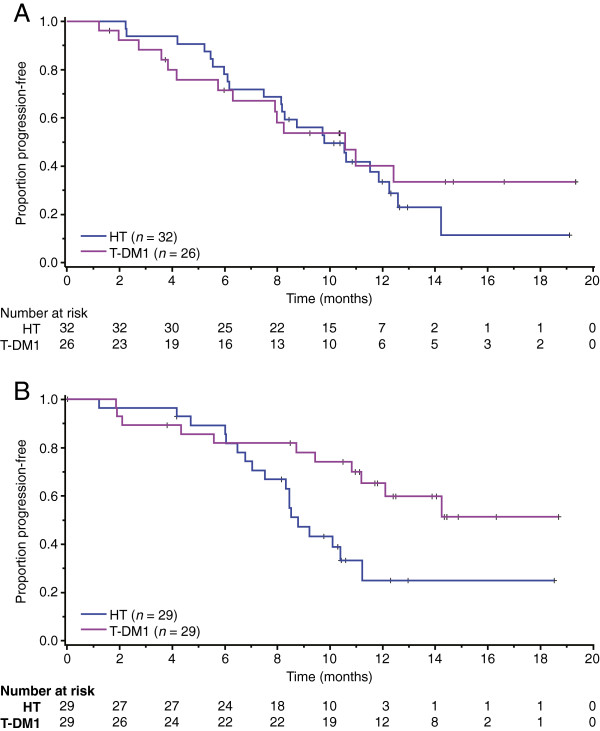
**Kaplan-Meier estimates of progression-free survival (PFS) by HER2 mRNA expression below the median and equal to or greater than median. (A)** Kaplan-Meier estimates of PFS by investigator assessment in patients with expression of HER2 mRNA below the median. The hazard ratio for progression was 0.85 (95% CI 0.44, 1.67). **(B)** Kaplan-Meier estimates of PFS by investigator assessment in patients with expression of HER2 mRNA equal to or greater than the median. The hazard ratio for progression was 0.39 (95% CI 0.18, 0.85). HER2, human epidermal growth factor receptor 2; HR, hazard ratio; HT, trastuzumab plus docetaxel; mRNA, messenger RNA; NR, not reached; T-DM1, trastuzumab emtansine.

**Figure 2:**
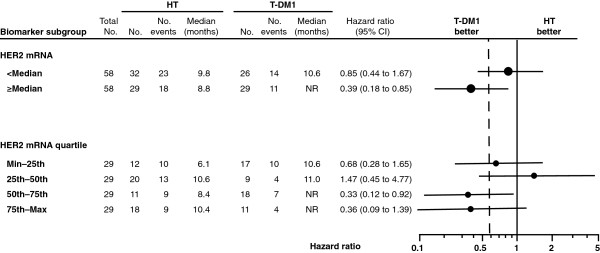
**Progression-free survival during first-line treatment by investigator assessment by biomarker subgroups.** The hazard ratios and 95% CIs for PFS are shown by HER2 mRNA subgroup (that is, below the median versus equal to or greater than the median, and by quartile). HER2, human epidermal growth factor receptor 2; HT, trastuzumab plus docetaxel; max, maximum; min, minimum; NR, not reached; T-DM1, trastuzumab emtansine.

**Figure 3:**
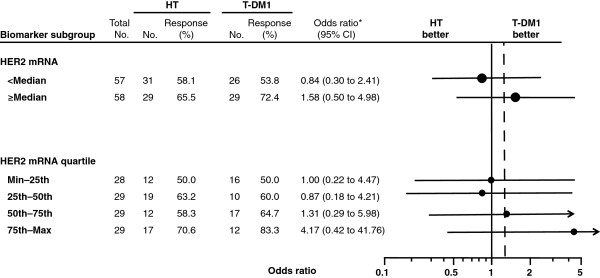
**Objective response during first-line treatment by investigator assessment by biomarker subgroups.** The odds ratios and 95% CIs for objective response rates are shown by HER2 mRNA subgroup (that is, below the median versus equal to or greater than the median, and by quartile). HER2, human epidermal growth factor receptor 2; HT, trastuzumab plus docetaxel; max, maximum; min, minimum; T-DM1, trastuzumab emtansine. *Odds ratio is defined as complete or partial response determined on two consecutive tumor assessments at least 4 weeks apart based on the response evaluation criteria in solid tumors [13]. Odds ratio relative to trastuzumab plus docetaxel was estimated by logistic regression. It was defined as the ratio of the odds of having a response in the T-DM1 arm and the odds in the trastuzumab plus docetaxel arm.

Efficacy outcomes (PFS and ORR) by HER2 mRNA expression level are summarized by treatment arm in Table 3. Kaplan-Meier estimates of PFS by HER2 mRNA expression for both treatment arms are presented in Figure 1A (expression below the median) and Figure 1B (expression equal to or greater than the median). The HRs/odds ratios and 95% CIs for PFS and ORR were calculated by HER2 mRNA subgroup (that is, equal to or greater than the median versus below the median, and by quartile) and are presented in Forrest plots in Figure 2 and Figure 3, respectively.

**Table 3:** Efficacy outcomes by tumor HER2 expression below the median and equal to or greater than the median

	**Median PFS, months**		
**HER2 expression**^**a**^	**HT**	**T-DM1**	**HR relative to HT (95% CI)**
All patients	(n = 70)	(n = 67)	0.59 (0.36, 0.97)
	9.2	14.2	
< Median	(n = 32)	(n = 26)	0.85 (0.44, 1.67)
	9.8	10.6	
≥ Median	(n = 29)	(n = 29)	0.39 (0.18, 0.85)
	8.8	Not reached	
	**ORR,%**		
**HER2 expression**^**a**^	**HT**	**T-DM1**	**Odds ratio relative to HT (95% CI)**
All patients	(n = 69)	(n = 67)	1.26 (0.63, 2.55)
	58.0	64.2	
< Median	(n = 31)	(n = 26)	0.84 (0.30, 2.41)
	58.1	53.8	
≥ Median	(n = 29)	(n = 29)	1.58 (0.50, 4.98)
	65.5	72.4	

^a^HER2 mRNA data were available for 61 of 70 patients in the HT arm and for 55 of 67 patients in the T-DM1 arm. HER2, human epidermal growth factor receptor 2; HT, trastuzumab plus docetaxel; ORR, objective response rate; PFS, progression-free survival; T-DM1, trastuzumab emtansine.

The risk of disease progression for all randomized patients was lower with T-DM1 than with HT (HR 0.59, 95% CI 0.36, 0.97) (Table 3). This effect was more pronounced in patients with tumor HER2 mRNA equal to or greater than the median (HR 0.39, 95% CI 0.18, 0.85) versus below the median (HR 0.85, 95% CI 0.44, 1.67). The magnitude of the difference in median PFS between the two treatment arms was greater in patients with tumors exhibiting HER2 expression equal to or greater than the median. Median PFS in patients with equal to or greater than the median HER2 expression was 8.8 months in the HT arm and was not reached in the T-DM1 arm (HR 0.39, 95% CI 0.18, 0.85). For patients with tumor HER2 expression below the median, median PFS was 9.8 months versus 10.6 months in the HT and T-DM1 arms, respectively (HR 0.85, 95% CI 0.44, 1.67).

In the T-DM1 treatment arm, median PFS was numerically longer in patients with HER2 expression equal to or greater than the median compared with those with HER2 expression below the median (not reached versus 10.6 months, respectively). In the HT arm, duration of PFS was similar in patients with HER2 expression equal to or greater than the median and below the median (8.8 versus 9.8 months, respectively).

In the overall patient population, the ORR in the T-DM1 arm was numerically greater than that in the HT arm (64.2% versus 58.0%; odds ratio 1.26, 95% CI 0.63, 2.55). For patients with tumor HER2 expression equal to or greater than the median, the ORR in the T-DM1 arm versus the HT arm was 72.4% versus 65.5% (odds ratio 1.58, 95% CI 0.50, 4.98). For patients with tumor HER2 expression below the median, the ORR was lower in the T-DM1 arm compared with the HT arm (53.8% versus 58.1%; odds ratio 0.84, 95% CI 0.30, 2.41).Results from the multivariate analyses indicated that after adjusting for baseline factors, including HER2 mRNA expression, there is a trend in PFS benefit for treatment with T-DM1 (HR 0.72, 95% CI 0.41, 1.27).

## Discussion

The TDM4450g (BO21976) study showed that treatment with single-agent T-DM1 as first-line treatment for MBC significantly improves PFS with a more favorable safety profile compared with combined HT treatment [10]. The PFS benefit was similar between the overall study population and in patients with centrally confirmed PFS in the primary analysis [10] as well as in this updated analysis (that is, data available from five additional patients) likely due to the relatively high concordance rate between local and central HER2 testing in this study. The retrospective and exploratory analysis reported here was carried out to investigate the relationship between the efficacy benefit of T-DM1 compared with HT and HER2 mRNA expression levels in TDM4450g. The results suggest that while T-DM1 provides clinical benefit in patients with HER2-positive tumors by standard criteria, the magnitude of efficacy of T-DM1 versus HT may be more pronounced in patients with tumors that exhibit HER2 mRNA expression above the median. This correlation suggests that high HER2 mRNA expression that results in higher expression of HER2 protein could lead to greater receptor presence on the cell surface that in turn may result in increased delivery of the cytotoxic agent DM1.

The results obtained from our analysis also highlight the importance of using the best available test to establish HER2 status. At present, patients are selected for HER2-targeted treatment based on the results of HER2 testing of their primary tumor, which is most commonly determined using either IHC or FISH [15]. In both cases, the result of HER2 testing is given as either positive or negative, and patients with HER2-positive tumors are considered to be eligible for treatment with an HER2-targeted agent. It is therefore imperative that individuals conducting HER2 testing ensure that the results they obtain are as accurate as possible. Our results suggest that qRT-PCR may be able to provide a more quantitative assessment of HER2 expression that could be used in addition to the standard tests for determining tumor HER2 status.

The findings from our exploratory analysis are hypothesis-generating only and should be interpreted with caution because of the limitations in the design of study TDM4450g. For example, the sample size was relatively small for each of the subgroups (fewer than 33 patients for all subgroups). These subgroup analyses are not statistically powered to detect a difference, their main purpose being to evaluate consistency of the effect across subgroups and to generate hypotheses to be tested in phase III studies. The study may also have been subject to bias, being open-label in design with investigator-assessed end points. Nevertheless, a potential association between HER2 mRNA expression and response to therapy has been reported in two phase II studies of single-agent T-DM1 [11,12]. In both of these studies, the median PFS was longer and the ORRs were greater in patients with HER2 mRNA expression at or greater than the median compared with those reported in patients with HER2 mRNA expression below the median. It is important to bear in mind, however, that both of those studies were also relatively small (approximately 25 to 35 patients per HER2 mRNA group) and had single-arm designs. Finally, in the majority of patients, HER2 mRNA was determined on tissue from the primary tumor. HER2 expression may have changed in the metastatic lesions. Such changes in HER2 expression have been documented [16,17]. However, these changes in HER2 expression appear to occur in a small number of patients (10% to 15%) [16,17]. We were unable to assess the potential influence of changes in HER2 expression in our study because of the small number of patients with tissue samples from metastatic sites.

The provocative data obtained in our study require validation in phase III trials. Preliminary results from EMILIA (TDM4370g/BO21977) presented at the American Association for Cancer Research suggest that the magnitude of the OS benefit with T-DM1 versus lapatinib plus capecitabine may be correlated with HER2 mRNA level [18]. Further analyses of HER2 mRNA expression in EMILIA, as well as in the ongoing phase III MARIANNE (TDM4788g/BO22589) and TH3RESA (TDM4997g/BO25734) trials, will help to evaluate further the correlation between this marker and clinical outcomes.

## Conclusions

In this exploratory analysis, we analyzed the relationship between tumor HER2 mRNA expression level and efficacy in patients receiving HT or T-DM1 in a randomized phase II study. While patients treated with T-DM1 had a lower risk of disease progression relative to HT-treated patients regardless of whether their tumors expressed high (equal to or greater than the median) or low (below the median) HER2 mRNA, the benefit was more pronounced in patients with high HER2 mRNA. If validated in phase III studies, these results suggest that there may be a subgroup of patients with an exceptional response to T-DM1 based on high HER2 mRNA expression levels.

## Abbreviations

ECOG PS: Eastern Cooperative Oncology Group performance status; ER: estrogen receptor; FISH: fluorescence in situ hybridization; HER2: human epidermal growth factor receptor 2; HR: hazard ratio; HT: trastuzumab plus docetaxel; IHC: immunohistochemistry; MBC: metastatic breast cancer; mRNA: messenger RNA; ORR: objective response rate; OS: overall survival; PFS: progression-free survival; PR: progesterone receptor; qRT-PCR: quantitative reverse transcriptase polymerase chain reaction; T-DM1: trastuzumab emtansine.

## Competing interests

EP has no disclosures. SH has served as an unpaid scientific advisor and/or medical advisor to Genentech, Novartis, Boehringer Ingelheim, and OBI Pharmaceuticals. SH has received travel reimbursement for conferences from Genentech/Roche and Novartis. LA, KM, VN, and EG, are full-time employees of Genentech and own Roche stock. LG has served on advisory boards or acted as a consultant for Roche, Genentech, GlaxoSmithKline, Wyeth, Novartis, Eisai, Pfizer, Millennium Takeda, Sanofi Aventis, Boehringer Ingelheim, Biogen Idec, Astra Zeneca, Genomic Health, Celgene, BioScience, and Taiho. LG holds a European Patent Application N.12195182.6 and 12196177.5 “PDL-1 expression in anti-HER2 therapy”.

## Authors’ contributions

EP contributed to the conception and design of this manuscript, the collection and assembly of data, data analysis and interpretation, and the writing of the manuscript. SH contributed to the conception and design of this manuscript, data analysis and interpretation, and the writing of the manuscript. LA contributed to the analysis and interpretation of data, and the writing of the manuscript. KM contributed to the conception and design of this manuscript, the collection and assembly of data, data analysis and interpretation, and the writing of the manuscript. VN contributed to the conception and design of this manuscript, the collection and assembly of data, data analysis and interpretation, and the writing of the manuscript. EG contributed to the analysis and interpretation of the data, and the writing of the manuscript. LG contributed to the analysis and interpretation of the data, and the writing of the manuscript. All authors read and approved the final version of the manuscript.

## Authors’ information

Presented in part at the 2012 European Society for Medical Oncology Congress, Vienna, Austria, 28 September to 2 October 2012. EA Perez, SA Hurvitz, LC Amler, V Ng, E Guardino, L Gianni. Exploratory analysis of the relationship between HER2 expression (by qRT-PCR) and efficacy with first-line trastuzumab emtansine (T-DM1) versus trastuzumab plus docetaxel in a randomized phase 2 study of patients with HER2-positive MBC. Poster 226P.

## Supplementary Material

Additional file 1 List of institutional review boards and participating study sites. Click here for file

Additional file 2 **CONSORT flow diagram.** Diagram depicting the flow of patients through study TDM4450g. Adapted with permission from Hurvitz *et al*. [10]. DFI, disease-free interval; HER2, human epidermal growth factor receptor 2; IV, intravenous; MBC, metastatic breast cancer; PD, disease progression; PFS, progression-free survival; ORR, objective response rate; q3w, once every 3 weeks; T-DM1, trastuzumab emtansine. Click here for file
