# Supplementary material for: Relationship between HER2 expression and efficacy with first-line trastuzumab emtansine compared with trastuzumab plus docetaxel in TDM4450g: a randomized phase II study of patients with previously untreated HER2-positive metastatic breast cancer
Source: Breast Cancer Res. 2014 May 23;16(3):R50. doi: 10.1186/bcr3661 (PMC4229898; doi:10.1186/bcr3661)
Supplement: Additional file 2 — CONSORT flow diagram. Diagram depicting the flow of patients through study TDM4450g. Adapted with permission from Hurvitz et al. [10]. DFI, disease-free interval; HER2, human epidermal growth factor receptor 2; IV, intravenous; MBC, metastatic breast cancer; PD, disease progression; PFS, progression-free survival; ORR, objective response rate; q3w, once every 3 weeks; T-DM1, trastuzumab emtansine. [file bcr3661-S2.pdf]

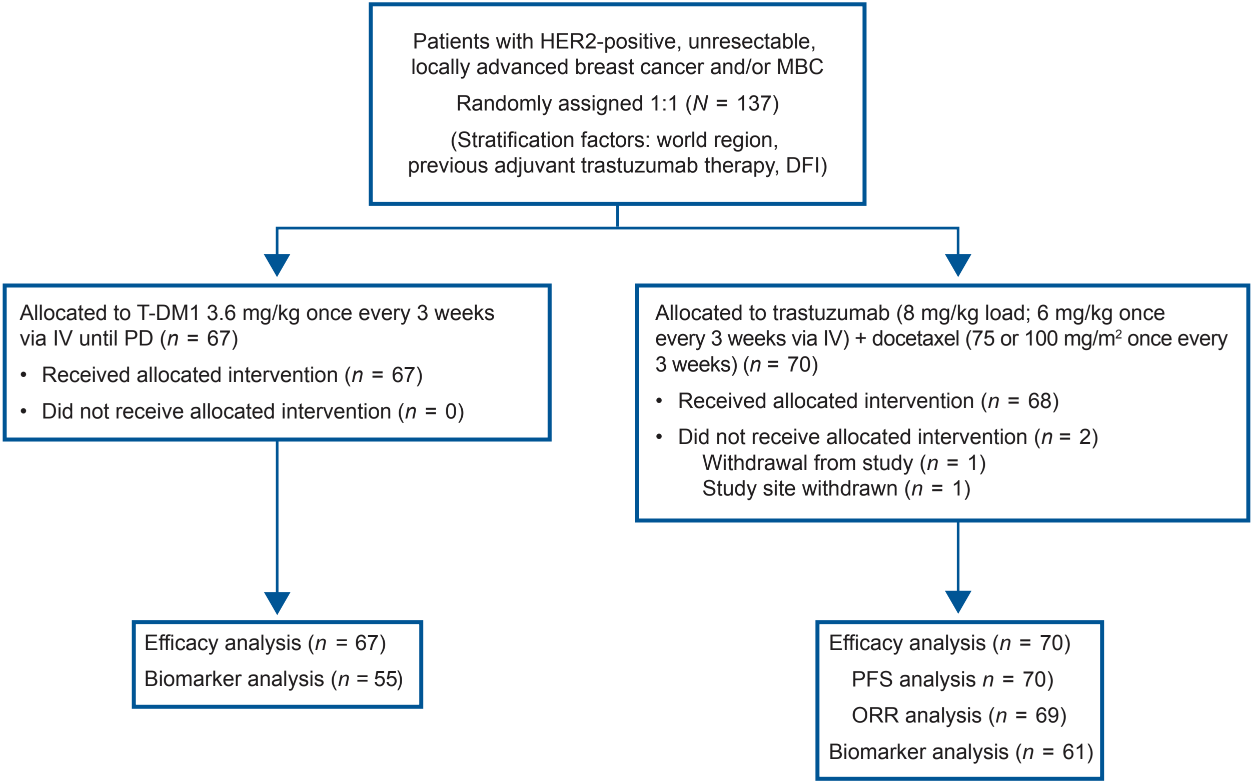

Patients with HER2-positive, unresectable, locally advanced breast cancer and/or MBC
Randomly assigned 1:1 (*N* = 137)
(Stratification factors: world region, previous adjuvant trastuzumab therapy, DFI)
Allocated to T-DM1 3.6 mg/kg once every 3 weeks via IV until PD (*n* = 67)
• Received allocated intervention (*n* = 67)
• Did not receive allocated intervention (*n* = 0)
Allocated to trastuzumab (8 mg/kg load; 6 mg/kg once every 3 weeks via IV) + docetaxel (75 or 100 mg/m^2 once every 3 weeks) (*n* = 70)
• Received allocated intervention (*n* = 68)
• Did not receive allocated intervention (*n* = 2)
Withdrawal from study (*n* = 1)
Study site withdrawn (*n* = 1)
Efficacy analysis (*n* = 67)
Biomarker analysis (*n* = 55)
Efficacy analysis (*n* = 70)
PFS analysis *n* = 70)
ORR analysis (*n* = 69)
Biomarker analysis (*n* = 61)
